# Interstitial lung disease in Primary Sjögren's syndrome

**DOI:** 10.1186/s12890-022-01868-5

**Published:** 2022-02-27

**Authors:** Wei Lin, Zhifei Xin, Jianlong Zhang, Ning Liu, Xiuying Ren, Meilu Liu, Yashuang Su, Yixuan Liu, Liu Yang, Shaoying Guo, Yupeng Yang, Yang Li, Jingjing Cao, Xiaoran Ning, Jingjing Li, He Xue, Nannan Niu, Yingmin Chen, Fang Li, Lijun Sun, Xiaopeng Zhang, Fengxiao Zhang, Wen Zhang

**Affiliations:** 1 Department of Rheumatology and Immunology, Hebei General Hospital, Hebei, 050051 Shijiazhuang China; 2 Department of Thoracic Surgery, Hebei General Hospital, Hebei, 050051 Shijiazhuang China; 3 Department of Medical Imaging, Hebei General Hospital, Hebei, 050051 Shijiazhuang China; 4 Department of Stomatology, Hebei General Hospital, Hebei, 050051 Shijiazhuang China; 5 Department of Oncology, Hebei General Hospital, Hebei, 050051 Shijiazhuang China; 6 Department of Rheumatology, Peking Union Medical College Hospital, Chinese Academy of Medical Science and Peking Union Medical College, National Clinical Research Center for Dermatologic and Immunologic Diseases, State Key Laboratory of Complex Severe and Rare Diseases, Beijing, 100730 China

**Keywords:** Primary Sjögren syndrome, Interstitial lung disease, Warrick score, Risk factors, Raynaud's phenomenon

## Abstract

**Background:**

Interstitial lung disease (ILD) may cause life-threatening complications of primary Sjogren’s syndrome (pSS), and has a poor prognosis in terms of survival and quality of life. To date, few studies have investigated the risk factors for ILD detected by high-resolution computed tomography (HRCT) in pSS patients with or without respiratory symptoms.

**Methods:**

Data of 333 patients with newly diagnosed pSS were retrospectively analysed. Interstitial lung disease involvement was defined as typical abnormalities on HRCT and/or pulmonary function tests. Multivariate regression model was used to evaluate the association between interstitial lung disease and pSS characteristics.

**Results:**

Sixty-six patients (19.82%) were diagnosed with pSS-ILD. Ground glass opacities (87.88%) and septal/sub pleural lines (81.82%) were most frequent. Based on pulmonary high-resolution computed tomography, patients were divided into nonspecific (n = 42), usual (n = 20), lymphocytic interstitial pneumonia (n = 3) and cryptogenic organising pneumonia (n = 1) groups. There was a strong association between erythrocyte sedimentation rate (ESR)/C-reactive protein (CRP) and the HRCT-score. Pulmonary function tests revealed impaired diffusion capacity for carbon monoxide and total lung capacity, and coexistence of small airway lesions in pSS-interstitial lung disease. On logistic regression analysis, age, Raynaud’s phenomenon, lymphopenia, cough, dyspnoea and rampant dental caries were risk factors associated with pSS-interstitial lung disease.

**Conclusions:**

Interstitial lung disease involvement in pSS is a common clinical occurrence. The clinical manifestation is nonspecific and variable; Raynaud’s phenomenon and lymphopenia may predict its onset. pSS patients with advanced age, dry cough and dyspnoea should be systematically evaluated for ILD involvement and managed according to their symptoms.

**Supplementary Information:**

The online version contains supplementary material available at 10.1186/s12890-022-01868-5.

## Introduction

Primary Sjögren syndrome (pSS) is a progressive autoimmune disease characterised by mononuclear cell infiltration of the exocrine glands, presented as sicca symptoms. A complicated interaction of genetic, environmental, and hormonal factors could be involved in its pathogenesis. Aberrant activation of the innate and acquired immune systems, such as type I and II interferons [1, 2], B- [3, 4], follicular helper T- [5], T helper type 17- [6], and T regulatory-cell [7] responses play a key role, explaining the potential systemic involvement, including vasculitis, lung, renal, and neurological involvement.

Lung complications are the most common extraglandular manifestations of pSS [8, 9], including interstitial lung disease (ILD), small airway disease (SAD), and lymphoproliferative disorders [10, 11]. In general, pSS-related ILD is the most frequent lung involvement in pSS and its most common manifestations are nonspecific interstitial (NSIP), usual interstitial (UIP), lymphocytic interstitial pneumonia (LIP) and organising pneumonia (OP). ILD could lead to life-threatening complications, including ventilatory failure and secondary pulmonary hypertension [12–14], which provide a poor prognosis in terms of survival and quality of life [11, 15, 16]. Thus, early ILD detection is crucial in pSS patients. However, it remains controversial whether all pSS patients should undergo a systematic search for lung involvement.

Previous studies have reported various risk factors associated with pSS-ILD including advanced age, smoking history, male sex, the presence of antinuclear (ANAs), anti-La/SS-B, and anti-Ro/SS-A antibodies, lymphopenia, a history of Raynaud's phenomenon, digestive involvement, and a long course of disease progression [3, 6, 9–12]. However, in most studies, pulmonary involvement was defined as the presence of respiratory symptoms accompanied with altered pulmonary diagnostic test results. However, a significant proportion (23.81%) of pSS- ILD patients do not report any respiratory symptoms [17]. With the development of imaging technology, high-resolution computed tomography (HRCT) is more sensitive than plain radiography and pulmonary function tests (PFTs) [4–8], especially in asymptomatic patients. To date, few studies have investigated the risk factors for ILD detected by HRCT in pSS patients with or without respiratory symptoms.

Therefore, we performed this study to describe the baseline characteristics and investigate the risk factors for ILD in pSS patients. We retrospectively investigated various clinical aspects of ILD associated with pSS, indicated by HRCT and PFTs, and the differences in these findings between the HRCT-scan patterns. Manifestations were assessed and compared in pSS patients with or without ILD. We also examined the relationships among clinical findings and changes after performing HRCT and PFT examinations. Finally, based on the comparative results, predictive factors for ILD onset were further identified.

## Material and methods

### Study subjects

In total, 333 patients with new-onset pSS admitted to Hebei General Hospital in China from September 2016 to March 2019 were enrolled. Their data were retrospectively reviewed and reanalysed for fulfilling the 2002 American-European Consensus Group criteria [18]. Patients diagnosed with other connective tissue diseases, including systemic lupus erythematosus, systemic scleroderma, idiopathic inflammatory myositis, and mixed connective tissue disease, were excluded. Their immunological and clinical profiles and extraglandular involvement were analysed. The examined ILD variables included date of diagnosis, HRCT-scans, and PFT results. This study was approved by the Institutional Ethical Committee of Hebei General Hospital (approval number: 2016070). Written informed consent was obtained from all individual participants. The procedures performed in this study were completed in accordance with the standards specified in the Helsinki Bulletin and the Chinese Laboratory Research Guidelines.

### Data collection

Data regarding demographic features and clinical and laboratory findings (i.e., age, sex, disease duration, symptoms, and pathological findings of labial gland biopsies) were retrospectively collected at the time of initial pSS diagnosis. Data of laboratory findings were obtained from individual patients at their first visit to the hospital, including: erythrocyte sedimentation rate (ESR), C-reactive protein (CRP) level, white blood cell count, hemoglobin level, leukocytosis, platelet count, rheumatoid factors (RF), blood protein electrophoresis, levels of IgG, IgM and IgA. Autoantibody screening was performed for: antinuclear antibodies (ANA), anti-SSA/SSB antibodies, anticentromere antibodies (ACA) and anti-RNP antibodies. The pSS patients having ILD and ACA were carefully assessed, and the systemic sclerosis (SSc) patients were excluded according to the 2013 classification criteria for SSc [19].We also collected clinical information regarding their history of arthritis, chronic fatigue, Raynaud’s phenomenon, liver involvement (primary biliary cirrhosis or autoimmune hepatitis), interstitial renal disease, glomerulonephritis, lymphadenopathy, myalgia, arthralgia, and peripheral or central nervous system involvement.

The Eular Sjögren's syndrome disease activity index (ESSDAI) was calculated [20–22]. The ESSDAI measures disease activity in pSS patients. There are 12 domains associated with disease activity (score ranges from 0 to 3). Treatment received before and during the process of the disease was also evaluated.

### HRCT and PFT analysis

HRCT scan of the lungs was performed in all patients. Thin-section CT images (1-mm slice thickness) were acquired at full inspiration and end-tidal expiration with a high spatial frequency reconstruction algorithm (Siemens Somatom Definition 128 slice CT system; Siemens Corp., Munich, Germany). The HRCT findings were categorised according to the CT patterns classification described by the American Thoracic Society/European Respiratory Society International Multidisciplinary Consensus Classification of the Idiopathic Interstitial Pneumonias [23]. A Warrick score was used to assess radiographic ILD severity and extent [24]. The total score varies between 0 and 30 and the higher score indicates a higher degree of radiological change. All image evaluations were performed by two radiographic specialists. All data were available. The PFT results were expressed as percentages of the predicted value of each parameter and corrected for sex, age, and height. The following parameters were evaluated at ILD diagnosis: vital capacity (VC), forced VC (FVC), and diffusing capacity for carbon monoxide (DLCO). Lung function was considered abnormal when volumes were < 80% of the predicted values and when DLCO was < 70% of the predicted value. FEF25-75 was evaluated to determine small airway function, and FEF25-75 < 65% was defined as abnormal.

### Statistical analysis

All statistical analyses were performed using SPSS version 25 software (IBM Corp., Armonk, NY). Data are described as frequencies for categorical variables and as the median and interquartile range (IQR) for quantitative variables. Two-group comparisons were analysed by Fisher’s exact test (two tailed) for categorical variables and by Student’s t test or Mann–Whitney U test for continuous variables according to Gaussian or non-Gaussian distribution, respectively. Chi-square tests were used to evaluate the differences in proportions. Correlations among the HRCT Warrick score, erythrocyte sedimentation rate (ESR), C-reactive protein (CRP), ESSDAI, and PFT were assessed with a Spearman’s rank correlation analysis using GraphPad Prism 8.0 (GraphPad Software Inc., San Diego, CA, USA). Univariate and backward stepwise multivariate logistic regression analyses were performed to identify risk factors for ILD involvement, and odds ratios (ORs) and 95% confidence intervals (CIs) were calculated. In the multivariable analysis, parameters with *p* values ≤ 0.1 in the univariate analysis were adjusted. For all analyses, the probability values were two-sided and *p* < 0.05 was considered to be significant.

## Results

### Demographic and clinical pSS-ILD characteristics

There were 333 patients with pSS (sex, 310 female [93.09%] and 23 male [6.91%]; median age at diagnosis, 54 years [range 18–85 years]). ILD was detected in 66 patients (19.82%) (eight men [12.12%] and 58 women [87.88%]). The data of the patients included in the pSS-ILD and non-pSS-ILD groups are summarised in Table 1. The patients with ILD were older (*p* = 0 0.01) than those without. A lower LMR (3.92 (2.50–5.16) vs. 5.41 (3.85–6.93), *p* < 0.001), higher NLR (2.46 (1.55–3.59) vs 1.95 (1.38–2.76), *p* = 0.01), and higher PLR (169.81 (111.46–248.69) vs 142.29 (109.63–196.84), *p* = 0.03) were observed in the ILD group. The level of ESR (23 (10.75–45.25) mm/1 h vs 17 (8–34) mm/1 h, *p* = 0.03) was higher in ILD group. An elevated CRP level (30.65% vs 17.46%, *p* = 0.02) was more frequently observed in patients with ILD. In addition, anti-RNP (30.30% vs. 7.12%; *p* < 0.001) and ACA (21.21% vs. 11.61%; *p* = 0.04) were more frequent in ILD patients. Moreover, the positive ANA rate was not significantly different between the two groups. Similarly, we found no statistically significant differences in CRP, serum C3 and C4 levels, positive rheumatoid factor, positive anti-SSA/Ro or anti-SSB/La antibodies, and immunoglobulin levels. Meanwhile, a focus score of ≥ 1 at histological evaluation of the minor salivary gland showed no between-group difference (92.06% vs. 96.54%; *p* = 0.16).

**Table 1:** Baseline characteristics of enrolled pSS patients with and without ILD

	pSS with ILD (n = 66)	pSS without ILD (n = 267)	*p* value
**Demographic features**			
Gender (F/M)	58/8	252/15	0.10
Age at onset, year	58.02 ± 13.78	52.91 ± 13.16	0.01
Disease duration, months	60 (24–120)	48 (12–120)	0.12
**Laboratory findings**			
White blood cell counts (× 10^9^/L)	4.74 (3.99–6.51)	4.97 (3.93–6.23)	0.88
Neutrophils counts (× 10^9^/L)	2.96 (2.34–4.40)	2.93 (2.15–4.14)	0.41
Lymphocytes counts (× 10^9^/L)	1.23 (0.91–1.74)	1.54 (1.14–1.89)	0.005
Haemoglobin (× g/L)	117 (109–129.75)	122 (110–132)	0.16
Platelets counts (× 10^9^/L)	217 (171.25–272.75)	224 (179–268)	0.89
LMR	3.92 (2.50–5.16)	5.41 (3.85–6.93)	< 0.001
NLR	2.46 (1.55–3.59)	1.95 (1.38–2.76)	0.01
PLR	169.81 (111.46–248.69)	142.29 (109.63–196.84)	0.03
ESR (mm/1 h)	23 (10.75–45.25)	17 (8–34)	0.03
CRP (mg/L)	3.3 (1.35–9.97)	3.3 (1.09–4.12)	0.24
RF(IU/L)	14.5 (10.6–63.55)	18.0 (10.6–69.0)	0.47
IgG(g/L)	14.85 (12.12–19.66	15.43 12.51–20.08	0.62
IgA(g/L)	2.44 1.87–3.42	2.83 1.98–3.70	0.07
IgM(g/L)	1.03 0.74–1.58	1.20 0.82–1.62	0.23
C3 (mg/L)	1.04 0.92–1.24	1.07 0.91–1.19	0.81
C4 (mg/L)	0.20 0.16–0.24	0.19 0.15–0.24	0.26
Elevated ESR (n, %)	33/62, 53.23	118/259, 45.56	0.28
Elevated CRP (n, %)	19/62, 30.65	44/252, 17.46	0.02
Low C3 (n, %)	14, 21.21	61, 22.85	0.78
Low C4 (n, %)	4, 6.06	25, 9.36	0.39
Elevated IgG (n, %)	20/65, 30.77	98/262, 37.40	0.32
RF (+) (n, %) *	26/59, 44.07	124/252, 49.21	0.48
ANA (+) (n, %) **	55, 83.33	208, 77.90	0.33
Anti-RNP (+) (n, %)	20, 30.30	19, 7.12	< 0.001
Anti-Ro52 (+) (n, %)	35, 53.03	165, 61.80	0.19
Anti-Ro/SSA (+) (n, %)	30, 45.45	157, 58.80	0.05
Anti-La/SSB(+) (n, %)	10, 15.15	69, 25.84	0.07
ACA (+) (n, %)	14, 21.21	31, 11.61	0.04
Pathological MSG with focus score ≥ 1 (n, %)	58/63, 92.06	251/260, 96.54	0.16
ESSDAI	15 (10.75–21)	7.0 (2–11)	< 0.001

*pSS* primary Sjögren’s syndrome, *RF* rheumatoid factor, *ANA* antinuclear antibodies, *MSG* minor salivary gland, *ACA* anti-centromere antibodies, *ESR* erythrocyte sedimentation rate, *IgM* immunoglobulin M, *IgA* immunoglobulin A, *IgG* immunoglobulin G, *CRP* C-reactive protein, *LMR* lymphocyte to monocyte ratio, *NLR* neutrophil to lymphocyte ratio *PLR* platelet to lymphocyte ratio, *MSG* minor salivary gland

^*^Positive RF > 20 IU/mL; **positive for ANA titres ≥ 1:320

ILD onset was identified to be concurrent with pSS in 13 (19.70%) patients and developed after pSS in 39 (59.09%), whereas in 14 (21.21%) patients the onset was prior to pSS. Pulmonary symptoms included dry cough (n = 32), sputum production (n = 23), chest tightness (n = 14), dyspnoea (n = 19), fever (n = 19), and recurrent pulmonary infections (n = 16) at ILD diagnosis. Additionally, 24 patients without obvious pulmonary symptoms exhibited abnormalities, consistent with ILD symptoms, in HRCT-scan and PFT results.

Initial clinical symptoms in pSS-ILD were nonspecific, and patients more commonly presented with cough (25.76% vs. 1.87%; *p* < 0.001), sputum production (10.61% vs. 0.75%; *p* < 0.001) and dyspnoea (19.70% vs. 1.87%; *p* < 0.001). Moreover, patients with ILD commonly exhibited Raynaud's phenomenon (18.18% vs. 2.62%; *p* < 0.001) and rampant dental caries (27.27% vs. 8.99%; *p* < 0.001), as the first symptom. Regarding the presence of dry mouth, dry eye, and other initial symptoms of pSS, we found no statistically significant difference between patients with and without ILD.

**Fig. 1:**
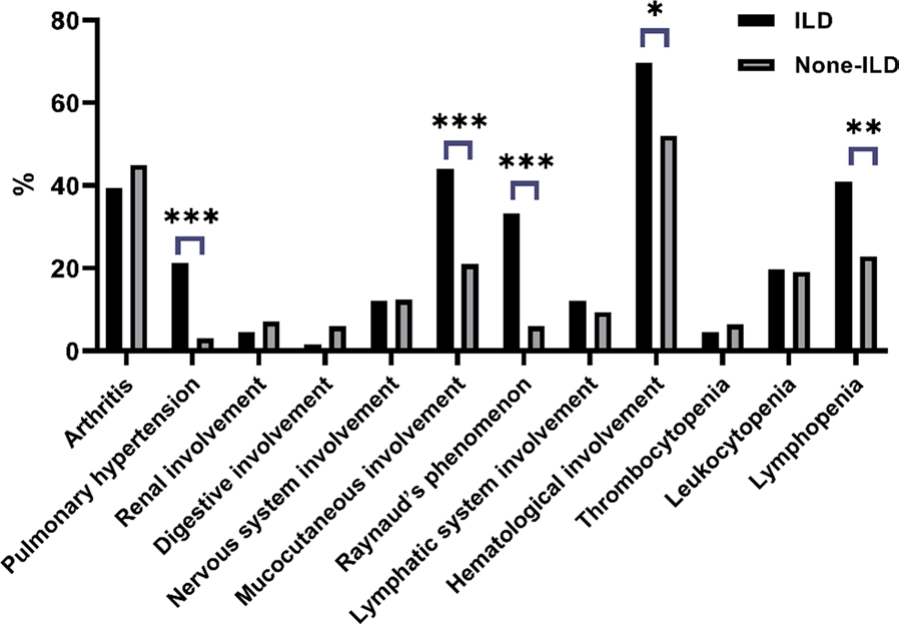
Extraglandular involvements in pSS patients with and without ILD

Compared to those without ILD, pSS-ILD patients had significantly higher prevalence of mucocutaneous involvement (43.94% vs. 20.97%, *p* < 0.001). Raynaud's phenomenon was observed in 22 (33.33%) patients with ILD, which was a significantly higher rate compared to those without (5.99%, *p* < 0.001). Haematological involvement was the main extraglandular manifestation of patients with ILD (46 out of 66 patients [69.70%]). Twenty-six (39.39%) patients suffered from musculoskeletal involvement, including arthralgias and transient synovitis. The neurologic manifestation, characterised by peripheral or cranial neuropathy, was observed in eight (12.12%) patients. Renal manifestation, including distal renal tubular acidosis and interstitial nephritis, was the less frequent symptom in patients with ILD (observed in only 3 patients [4.55%]) (Fig. 1). The ESSDAI score of pSS-ILD patients was significantly higher (15 (10.75–21)) compared to those without (7.0 (2–11), *p* < 0.001). Additionally, investigation of the association between the ESSDAI and total HRCT-scores revealed a strong positive correlation (r = 0.28, *p* = 0.03).

All patients with pSS-ILD received prednisolone, immunosuppressants, or a combination of both. During the follow-up period (mean follow-up time, 34.26 ± 5.77 months), except for 5 patients without regular follow-up, one of the 61 patients died due to acute exacerbation of ILD, 3 patients developed SLE, and one patient suffered recurrent pulmonary infections.

### HRCT findings and pulmonary function of patients with ILD

Lung HRCT-scan findings demonstrated the following abnormalities: ground glass opacities (n = 58; 87.88%), irregularities in pleural margins (n = 47; 71.21%), septal/sub pleural lines (n = 54; 81.82%), honeycombing appearance (n = 19; 28.79%), and sub pleural cysts (n = 27; 40.90%). Additionally, NSIP was the predominant pulmonary HRCT pattern (n = 42, 63.64%; Table 2). Besides, lung HRCT findings revealed UIP, LIP and COP patterns in 20 (30.30%), three (4.55%) and 1(1.52%) patients, respectively.

**Table 2:** Characteristics of ILD in 66 patients with pSS

	ILD-onset
**Time of ILD onset (n, %)**	
Before pSS onset	14, 21.21%
Concomitant with pSS	13, 19.70%
After pSS onset	39, 59.09%
**Pulmonary symptoms (n, %)**	
Dry cough	32, 48.48%
Sputum production	23, 34.85%
Chest tightness	14, 21.21%
Dyspnea	19, 28.79%
Fever	19, 28.79%
Recurrent pulmonary infections	16, 24.24%
**Baseline pulmonary function**	
TLC, % predicted	74.32 ± 17.27
FVC, % predicted	82.50 (71.25–97)
FEV1, % predicted	84 (71–96.75)
DLCO, % predicted	58.82 ± 21.04
FEV1/FVC	100 (95.25–103)
RV, % predicted	72.24 ± 24.09
RV/TLC	94.87 ± 17.56
FEF25, % predicted	74.4 ± 28.59
FEF50, % predicted	64.30 ± 24.20
FEF75, % predicted	61.07 ± 27.76
**HRCT findings (n, %)**	
Ground glass opacities	58, 87.88%
Irregularities in pleural margins	47, 71.21%
Septal/sub pleural lines	54, 81.82%
Honeycombing appearance	19, 28.79%
Sub pleural cysts	27, 40.90%
parenchymal micronodules/nodules	61, 92.42%
**HRCT scan pattern (n, %)**	
NSIP	42, 63.64%
UIP	20, 30.30%
LIP	3, 4.55%
COP	1, 1.52%
**HRCT CoVR quantification**	
HRCT extent	6 (4–9)
HRCT severity	6 (5–15)
HRCT total score	12 (9–24)

Among the 66 patients with ILD, 40 had recorded PFTs (Table 2). At the time of ILD diagnosis, diffusion capacity impairment was the predominant manifestation of pulmonary involvement (n = 30, 75%) and the most severe complication with a predicted DLCO value of 58.82 ± 21.04%. Moreover, PFTs indicated severe impairment with a predicted DLCO values < 50% in 25% of patients (n = 10). Notably, impaired small airway resistance (n = 14, 35%) was a more common incidence in this series. Additionally, it was demonstrated that 40% (n = 16) and 27.5% (n = 11) of the patients had impaired ventilatory function and restrictive disease pattern. Furthermore, 2 (5%) patients showed a severe impairment with a predicted FEV1 values < 35%.

### HRCT findings and pulmonary function of ILD patients with UIP and NSIP

In this instance, we observed that ILD patients, who had UIP tended to exhibit higher Warrick score (25 [24–29.5] vs. 10.5 [8–12], *p* < 0.001), a lower predicted FEV1 value (72 [59.75–85.50] vs. 93.5 [81.25–99], *p* = 0.001) and lower predicted FVC (71 [65.25–83.0] vs. 88 [80.5–102.75], *p* = 0.001) value at ILD diagnosis compared with those having NSIP. We also found that patients with UIP exhibited significantly lower predicted DLCO values (43.07 ± 19.26% vs. 69.09 ± 16.49%, *p* < 0.001) at ILD diagnosis. Hence, the pSS-ILD patients with UIP patterns were further associated with diffusive and restrictive ventilation dysfunction. Additionally, the patients from both groups suffered from SAD (FEF50, % predicted: 49.71 ± 22.32% vs 72.92 ± 21.31%, *p* = 0.003; FEF75, % predicted: 54.64 ± 31.55% vs 66 ± 24.34%, *p* = 0.22; FEF25-75, % predicted: 68.43 ± 28.41% vs 89.58 ± 26.35%, *p* = 0.03; respectively) (Additional file 1: Table S1).

### Correlation between the HRCT-score and PFT results

**Fig. 2:**
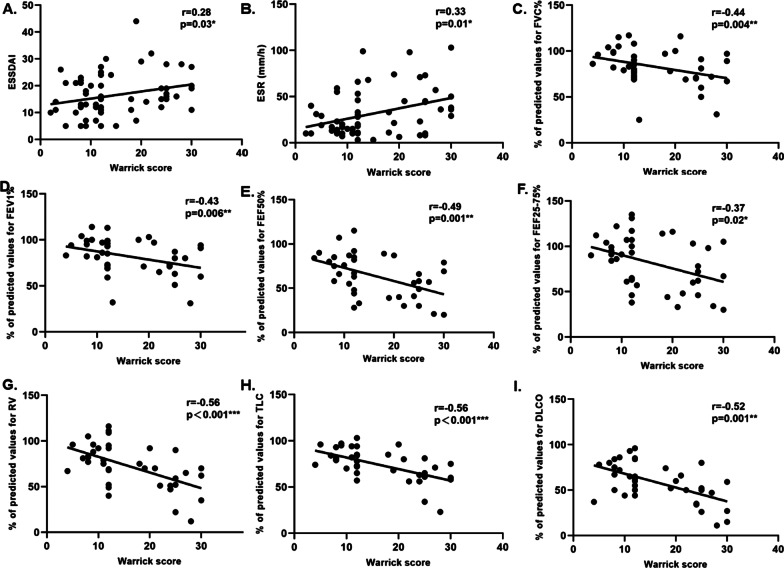
Correlation between the HRCT-score and PFT results in pSS-ILD patients

As shown in Fig. 2, investigation of the association between the total HRCT-scores and PFT results revealed strong negative correlations between the HRCT-scores and FVC (r = − 0.44, *p* = 0.004), FEV1 (r = − 0.43, *p* = 0.006), FEF25% (r = − 0.34, *p* = 0.03), FEF25-75% (r = − 0.37, *p* = 0.02), DLCO (r = − 0.52, *p* = 0.001), TLC (r = − 0.56, *p* < 0.001), and RV (r = − 0.56, *p* < 0.001). However, there were no correlations between the HRCT-scores and PEF 75% (r = − 0.22, *p* = 0.17). In addition, the total HRCT-scores showed a significant positively correlation with ESSDAI (r = 0.28, *p* = 0.03), ESR (r = 0.33, *p* = 0.01) and CRP(r = 0.41, *p* = 0.001).

### Potential risk factors for pSS-ILD

**Fig. 3:**
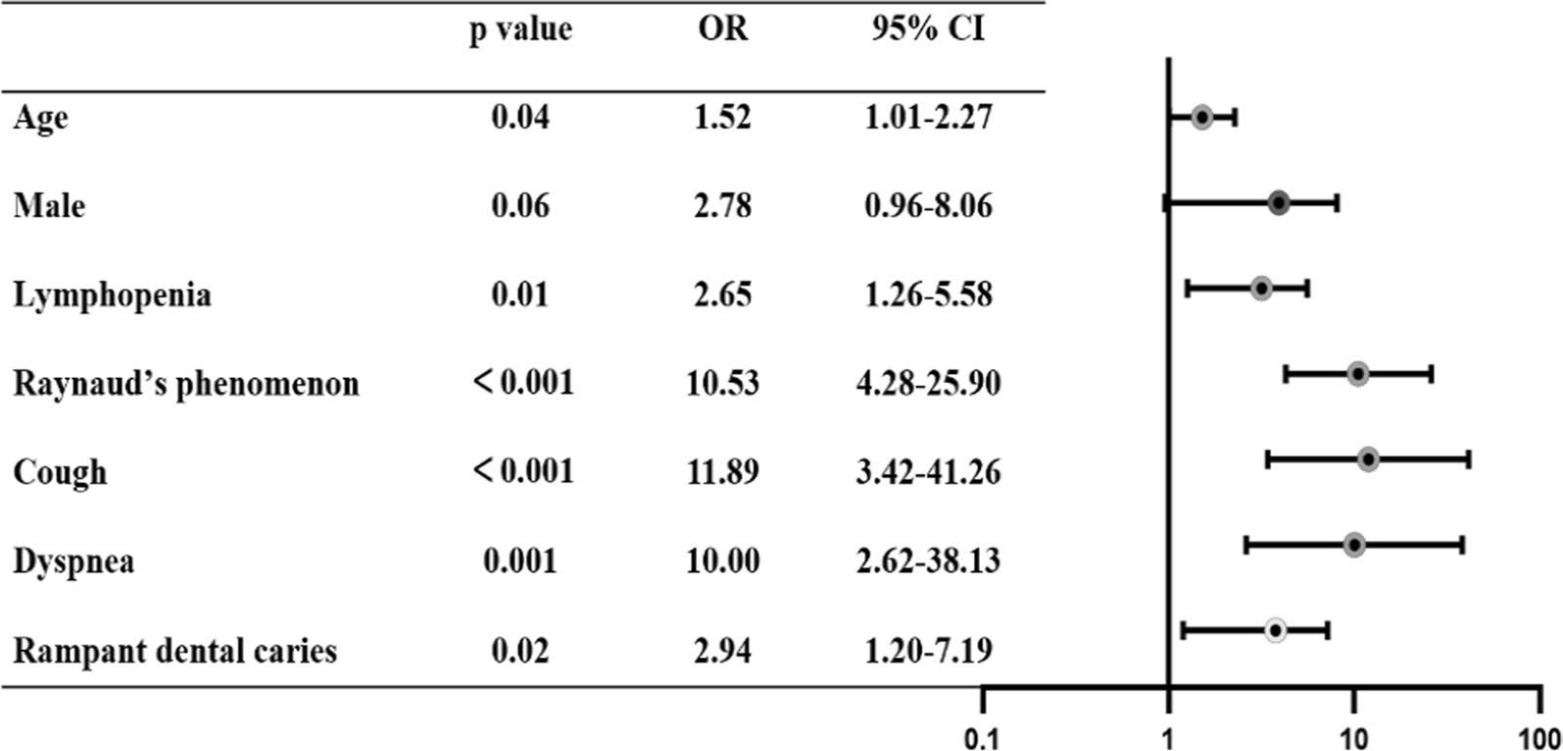
Multivariate analysis of features predicting ILD in pSS patients. *CI* Confidence interval, *OR* odds ratio

The multivariable analysis found that age (*p* = 0.04; OR 1.52; 95% CI 1.01–2.27), Raynaud’s phenomenon (*p* < 0.001; OR 10.53; 95% CI 4.28–25.90), lymphopenia (*p* = 0.01; OR 2.65; 95% CI 1.26–5.58), cough (*p* < 0.001; OR 11.89; 95% CI 3.42–41.26), dyspnoea (*p* = 0.001; OR 10.00; 95% CI 2.62–38.13) and rampant dental caries (*p* = 0.02; OR 2.94; 95% CI 1.20–7.19) were risk factors for ILD. These data are presented in Fig. 3.

## Discussion

In this study, we retrospectively analysed the clinical characteristics of 333 Chinese pSS patients and demonstrated that the prevalence of ILD was 19.82%. Moreover, we showed that dry cough, chest tightness, and Raynaud's phenomenon were the most significant predictive factors. To the best of our knowledge, this is the first study that showed the prevalence, clinical characteristics, HRCT-score, and outcome of different ILD pattern in Chinese patients with pSS.

Evidence regarding the prevalence of ILD in patients with pSS is controversial. Previous studies have reported a prevalence of pSS-ILD ranging from 1.01 to 78.61% [11, 12, 17, 25–29]. This wide range is partially attributed to the different study design, background population, case selection, and improved diagnostic techniques. In our total patient cohort, the prevalence of ILD manifestations was 19.82%, consistent with those reported in other studies [14, 28, 29]. Interestingly, we detected a significantly higher pSS-ILD prevalence in Asia than in Europe. These data suggested differences in the pathogenesis of patients with pSS with different geographic and ethnic backgrounds. Li Y et al. reported that pSS patients from different ethnicities and locations possess distinct susceptibility locus [30], which might modulate the clinical phenotypes. Additionally, Nannini C has previously underlined that the cumulative incidence of ILD in patients with pSS was 10% one year after pSS diagnosis and increased to 20% five years after initial onset [31], suggesting that the patients with pSS-ILD require a closer follow-up.

Besides, in 14 (21.21%) patients, pulmonary involvement was the initial manifestation of pSS and even preceded the diagnosis, which was consistent with previous studies [17, 32]. In a prospective study, ILD was the main presenting symptom in 13 (16.88%) newly diagnosed patients with pSS [28], while typical autoantibodies were often absent. Further, a multicentre study detected 25 patients with pSS-ILD and in 60% of them, the ILD diagnosis preceded that of pSS [32]. Clinical manifestations of lung involvement in patients with pSS can be nonspecific, therefore, delay diagnosis. Therefore, these features underlined the importance of performing differential diagnosis in the initial disease stage and multidisciplinary communication.

HRCT is considered a helpful and sensitive method for detecting lung abnormalities in patients with pSS. HRCT-scan was useful in identifying ILD presentations, with linear (81.82%), ground-glass opacities (87.88%) and parenchymal micronodules/nodules (92.42%) being the most common abnormalities in our series. Previous studies have reported that ground-glass and non-septal linear opacities were more commonly exhibited on HRCT-scan of pSS patients [13, 33, 34]. Interestingly, we detected a preponderance of micronodules/nodules in pSS-ILD patients, probably because improved diagnostic technology may have resulted in a higher sensitivity for the detection of lung nodules. Moreover, multiple nodules suggested an inflammatory role in the disease pathogenesis. Besides, pSS patients with multiple lung nodules tend to have increased risk of lymphomas, especially in the mucosa-associated lymphoid tissue [35]. Our study confirmed that HRCT-scan is supportive for pSS-ILD histological pattern identification, as previous studies suggested [17]. Our analysis of the HRCT-scans indicated that pSS patients mostly suffered from NSIP (63.64%); these findings are consistent with a previous study that examined 201 pSS patients showing a preponderance of NSIP (n = 72, 45.5%), UIP (n = 16, 10.10%), and LIP (n = 13, 8.2%) [26]. Although LIP rarely occurs, pSS is its most common related disease. Therefore, patients with LIP should consider undergoing pSS disease screening. In our study, only three (4.55%) pSS-ILD patients exhibited LIP. In 15 pSS-ILD cases in the literature, Dong et al. evaluated the onset of LIP, which is usually more insidious [36]. Further, to the best of our knowledge, our work was the first to analyse the association between the HRCT-score and the different ILD patterns and showed that the patients with UIP had a higher HRCT-score. Interestingly, the observed strong association between ESR/CRP and HRCT-score in our study suggested a higher disease activity in the ILD patients with pSS.

In our study, patients with pSS-ILD had abnormal PFT results, which were mainly associated with diffusive dysfunction and a restrictive pattern of abnormality. These findings were consistent with the results of previous studies [10, 37, 38]. Reduced DLCO seemed to be the most common abnormality [10, 39]. Moreover, patients with ILD (35%) also suffered from SAD. Dong et al. found that the rate was higher in patients with pSS-ILD than in those without ILD [34]. Previous studies have also reported that concomitant SAD was common in biopsy of proven pSS-ILD [39, 40]. There are limited studies focusing on the relationship between the HRCT-score and PFT. This study revealed that impairment of DLCO/RV/TLC was associated with high HRCT-scores, which was similar to the findings of previous studies reported by Chen et al. and Yazisiz et al., which also indicated that the HRCT-scores had a negative correlation with DLCO alone [41, 42]. Taken together, we postulated that higher HRCT-scores are correlated with more severe lung function impairment.

As ILD has been considered to be associated with premature mortality [15, 31], identification of risk factors for these diseases is crucial. Our results showed that advanced age and male sex were risk factors for pSS-ILD patients similar to previous findings [14, 17, 25, 26]. In contrast, there were no significant differences observed regarding the disease duration between pSS patients with and without ILD. The prevalence of dry cough and chest tightness was significantly higher as initial symptoms of ILD in patients and predicted its onset. Most patients with pSS (41–66%) suffered from cough [17]. Moreover, dry cough may present several years before the diagnosis of pSS and affects the quality of life in approximately 50% of pSS patients [43]. The cause of dry cough remains unclear. In general, dry cough is considered as the manifestation of SAD, which suggests that the coexistence of the two conditions is likely rather than constant. Airway dryness, bronchial or bronchiolar inflammation, hyper-reactive airway, and gastro-oesophageal reflux have proposed to explain coughing [17, 44].

Interestingly, patients ILD presented with Raynaud’s phenomenon more frequently compared with those without ILD, in line with previous reported findings [17, 27]. Roca et al. suggested a higher prevalence of Raynaud's phenomenon in pSS-ILD patients demonstrated that an ischaemic process may play a role in the lung damage onset [17]. Our data reinforced the hypothesis that microangiopathy may become integral for early detection of pSS patients at risk for ILD. Patients with pSS with RP require a closer monitoring of lung parameters (HRCT-scan and PFTs). Moreover, it is questionable whether a more aggressive therapy of RP in pSS patients would improve the course of ILD or prevent its onset. The underlying pathogenic mechanism of the relationship between RP and ILD should be studied further.

However, our study had some limitations. First, this was a retrospective, observational study; therefore, the causative relationship could not be well determined. Additionally, a small group of patients underwent PFTs, which was a limiting factor for generalising our conclusions, as the data of patients without PFTs could not be analysed to evaluate their lung or airway impairments. Therefore, important information could have been missed. Moreover, this was a single-centre study. Further, it was occasionally difficult to discriminate NSIP from UIP and LIP on HRCT without pathologic evaluation. Furthermore, we did not have a right heart catheterization in all patients with elevated pulmonary arterial pressure on ultrasound, we were not able to differentiate well whether PH was pre- or postcapillary hypertension. What’s more, among patients with pSS, pulmonary hypertension can occur in isolation (PAH) or in association with ILD (PH-ILD). Therefore, to make the results more reliable, although the proportion of patients with pulmonary arterial hypertension in the ILD group was significantly higher in the univariate analysis, it was considered inappropriate to perform a multivariate logistic regression analysis on the pulmonary arterial hypertension data to identify risk factors for ILD involvement. A prospective, large cohort study should be conducted to confirm our findings.

In conclusion, ILD involvement in pSS cases is a prevalent clinical occurrence. The clinical manifestations vary and are nonspecific. Accordingly, initial symptoms can be vague, and dry cough and dyspnea can predict ILD occurrence. Advanced age and Raynaud's phenomenon are risk factors for pulmonary interstitial complications in pSS patients. These patients should be systematically evaluated for ILD involvement and managed according to their symptoms. As the pathogenesis of pSS-ILD is complex, it is necessary to clarify the potential molecular biological mechanism between these risk factors and pSS-ILD in a large multicentre prospective study.

## Supplementary Information

**Additional file 1: Sup Table 1.** HRCT score and pulmonary function of ILD patients with UIP and NSIP.

## Data Availability

All data relevant to the study are included in the article or uploaded as online supplementary information. The data that support the findings of this study are available from the corresponding authors on reasonable request.

## Abbreviations

pSS: Primary Sjögren's syndrome
CRP: C-reactive protein
ILD: Interstitial lung disease
SAD: Small airway disease
NSIP: Nonspecific interstitial
UIP: Usual interstitial
LIP: Lymphocytic interstitial pneumonia
OP: Organising pneumonia
ANA: Antinuclear
HRCT: High-resolution computed tomography
PFTs: Pulmonary function tests
ESSDAI: The Eular Sjögren's syndrome disease activity index
FEV1: Forced expiratory volume in the 1st s
FVC: Forced vital capacity
DLCO: Carbon monoxide-diffusing capacity
RV: Residual volume
PEF: Peak expiratory flow
SPSS Inc, Chicago, IL, USA: The Statistical Package for the Social Sciences, version 25.0
ESR: Erythrocyte sedimentation rate
ORs: Odds ratios
CIs: Confidence intervals
