# Supplementary material for: Interstitial lung disease in Primary Sjögren's syndrome
Source: BMC Pulm Med. 2022 Feb 27;22:73. doi: 10.1186/s12890-022-01868-5 (PMC8882286; doi:10.1186/s12890-022-01868-5)
Supplement: Supplementary file 1 — Additional file 1: Sup Table 1. HRCT score and pulmonary function of ILD patients with UIP and NSIP. [file 12890_2022_1868_MOESM1_ESM.docx]

|  | NSIP | UIP | p |
| --- | --- | --- | --- |
| Warrick score | (n=40) (n=20) | | |
| HRCT total score | 10.5 (8-12) | 25 (24-29.5) | <0.001 |
| HRCT extent | 4 (3-6) | 10 (9-14.5) | <0.001 |
| HRCT severity | 6 (4-6) | 15 (15-15) | <0.001 |
| Pulmonary function | (n=25) (n=15) | | |
| TLC, % predicted | 82.73±12.02 | 60±15.37 | <0.001 |
| FVC, % predicted | 88 (80.05-102.75) | 71 (65.25-83) | 0.001 |
| FEV1, % predicted | 93.5 (81.25-99) | 72 (59.75-85.50) | 0.001 |
| DLCO, % predicted | 69.09±16.49 | 43.07±19.26 | <0.001 |
| FEV1/FVC | 100 (96-103) | 100.5 (94.75-104.25) | 0.96 |
| RV, % predicted | 84.05±18.67 | 52.43±20.00 | <0.001 |
| RV/TLC | 99.55±14.88 | 86.79±20.20 | 0.04 |
| FEF25, % predicted | 84.29±26.19 | 58.21±26.64 | 0.006 |
| FEF50, % predicted | 72.92±21.31 | 49.71±22.32 | 0.003 |
| FEF75, % predicted | 66±24.34 | 54.64±31.55 | 0.22 |
| FEF25-75, % predicted | 89.58±26.35 | 68.43±28.41 | 0.03 |
| DL/VA | 81.95±11.63 | 60.36±16.36 | 0.053 |

**Sup Table 1.** HRCT score and pulmonary function of ILD patients with UIP and NSIP
